# Retracted: Antiulcerogenic Activity of Li-Zhong Decoction on Duodenal Ulcers Induced by Indomethacin in Rats: Involvement of TLR-2/MyD88 Signaling Pathway

**DOI:** 10.1155/2024/9831531

**Published:** 2024-07-25

**Authors:** Evidence-Based Complementary and Alternative Medicine

RETRACTION: H. Song, M. Zeng, X. Chen, X. Chen, J. Peng, Y. Lin, R. Yu, X. Cai, Q. Peng, “Antiulcerogenic Activity of Li-Zhong Decoction on Duodenal Ulcers Induced by Indomethacin in Rats: Involvement of TLR-2/MyD88 Signaling Pathway,” Evidence-Based Complementary and Alternative Medicine, (2020) 14 pages, https://doi.org/10.1155/2020/6538156.

The above article, published online on 22 January 2020, has been retracted by agreement of the Editorial Board and John Wiley & Sons Ltd [1]. The retraction has been agreed to due to significant similarities between Figure 3(a) ESO (4.17 mg/kg) and Figure 3(a) LZD (7.50 g/kg) as originally raised on PubPeer [2].

When requested, the authors provided their explanation and the raw images. They feel that it is conceivable for such similarities to occur, noting the differences in the intestinal glands, muscularis mucosa, and submucosa, and maintain that the images are correct. However, this did not satisfy our concerns and the Editorial Board recommended retraction.
